# High Uptake of Exclusive Breastfeeding and Reduced Early Post-Natal HIV Transmission

**DOI:** 10.1371/journal.pone.0001363

**Published:** 2007-12-26

**Authors:** Louise Kuhn, Moses Sinkala, Chipepo Kankasa, Katherine Semrau, Prisca Kasonde, Nancy Scott, Mwiya Mwiya, Cheswa Vwalika, Jan Walter, Wei-Yann Tsai, Grace M. Aldrovandi, Donald M. Thea

**Affiliations:** 1 Gertrude H. Sergievsky Center, and Department of Epidemiology, Mailman School of Public Health, Columbia University, New York, New York, United States of America; 2 Lusaka District Health Management Team, Lusaka, Zambia; 3 University Teaching Hospital, University of Zambia, Lusaka, Zambia; 4 Center for International Health & Development, Boston University School of Public Health, Boston, Massachusetts, United States of America; 5 Department of Biostatistics, Mailman School of Public Health, Columbia University, New York, New York, United States of America; 6 Department of Pediatrics, Children's Hospital Los Angeles, University of Southern California, Los Angeles, California, United States of America; Partners for Applied Social Sciences (PASS), Belgium

## Abstract

**Background:**

Empirical data showing the clear benefits of exclusive breastfeeding (EBF) for HIV prevention are needed to encourage implementation of lactation support programs for HIV-infected women in low resource settings among whom replacement feeding is unsafe. We conducted a prospective, observational study in Lusaka, Zambia, to test the hypothesis that EBF is associated with a lower risk of postnatal HIV transmission than non-EBF.

**Methods and Results:**

As part of a randomized trial of early weaning, 958 HIV-infected women and their infants were recruited and all were encouraged to breastfeed exclusively to 4 months. Single-dose nevirapine was provided to prevent transmission. Regular samples were collected from infants to 24 months of age and tested by PCR. Detailed measurements of actual feeding behaviors were collected to examine, in an observational analysis, associations between feeding practices and postnatal HIV transmission. Uptake of EBF was high with 84% of women reporting only EBF cumulatively to 4 months. Post-natal HIV transmission before 4 months was significantly lower (p = 0.004) among EBF (0.040 95% CI: 0.024–0.055) than non-EBF infants (0.102 95% CI: 0.047–0.157); time-dependent Relative Hazard (RH) of transmission due to non-EBF = 3.48 (95% CI: 1.71–7.08). There were no significant differences in the severity of disease between EBF and non-EBF mothers and the association remained significant (RH = 2.68 95% CI: 1.28–5.62) after adjusting for maternal CD4 count, plasma viral load, syphilis screening results and low birth weight.

**Conclusions:**

Non-EBF more than doubles the risk of early postnatal HIV transmission. Programs to support EBF should be expanded universally in low resource settings. EBF is an affordable, feasible, acceptable, safe and sustainable practice that also reduces HIV transmission providing HIV-infected women with a means to protect their children's lives.

**Trial Registration:**

ClinicalTrials.gov NCT00310726

## Introduction

Promotion of exclusive breastfeeding (EBF) has been a cornerstone of public health measures to promote child survival for several decades [1], [2]. EBF is associated with lower risks of diarrhea- and pneumonia-related infant morbidity and mortality than breastfeeding with addition of other fluids and solids in both developed and developing world settings [3]–[6]. EBF facilitates normal physiological regulation of milk production, which depends on regular infant suckling, allowing for a healthy balance between the infant's needs and the amount of milk produced [7]. This regulation helps prevent milk stasis that underlies the development of mastitis and other breast problems [8] thereby being the healthiest practice for both mothers and infants.

Despite the well-established benefits of EBF in the absence of HIV, initial findings from Durban, South Africa, that the risk of post-natal HIV transmission was lower with EBF than with non-EBF [9], [10] were met with some skepticism [11]–[13]. As with all epidemiologic associations based on health-related behaviors that cannot be randomly assigned, it was difficult to rule out reverse causality, i.e. mothers of infected children choosing non-EBF, rather than EBF reducing risks of HIV infection, and confounding, i.e. a third factor related to transmission and to the likelihood of non-EBF, as possible explanations for the findings. Nevertheless, the finding was soon confirmed by a second large study conducted in Zimbabwe [14] and by a new study in KwaZulu-Natal, South Africa [15].

The public health benefits of EBF in low resource settings could be substantial. EBF can be practiced by women regardless of their HIV status thus avoiding the stigma of distinguishing infant feeding practices by maternal serostatus. Any reduction of HIV transmission due to EBF, would simply add to its established benefits for other aspects of infant and maternal health. EBF is unlike replacement feeding where reduction of HIV transmission has to be carefully balanced against increased mortality due to other infections. Support for EBF programs would be strengthened by more rigorous data showing the clear benefits of EBF for HIV prevention. Here we present the results of a prospective, observational study in Lusaka, Zambia, designed to test the *a priori* hypothesis that EBF is associated with a lower risk of early postnatal HIV transmission than non-EBF.

## Methods

### Study design

We conducted a prospective, epidemiologic study nested within a randomized trial evaluating the safety and efficacy of early weaning [16]. The protocol is available as a supporting document Protocol S1. We ruled out an experimental design to address the EBF hypothesis as impractical and unethical since, although the role of EBF in prevention of HIV transmission was in question at the time the study was designed, the benefits of this practice for other aspects of maternal and child health were already well-established. All women were encouraged to breastfeed exclusively to 4 months and the study was designed with the *a priori* objective of analyzing associations between actual practices and early postnatal HIV transmission. We improved on previous studies by including: more frequent measurements of the infants' HIV status to more accurately determine the timing of transmission; detailed questionnaires about feeding practices including 24 hour recall and feeding history corroborated with counselors' assessments; and collection of data necessary to make statistical adjustments for possible confounding by major risk factors for HIV transmission, namely maternal CD4 count and viral load.

### Study population

HIV-infected women were recruited between May 2001 and September 2004 from prevention of mother-to-child transmission (PMTCT) programs established at two antenatal clinics in Lusaka, Zambia. The programs offered voluntary HIV counseling and testing with nevirapine prophylaxis [17]. HIV treatment programs were established in 2004 [18] whereafter consenting, eligible women at any stage of follow-up were started on first-line treatment regimens. HIV-seropositive women were eligible for enrollment if they intended to breastfeed, accepted nevirapine, had no severe pregnancy complications and agreed to requirements of the study. Cotrimoxazole was given to all women with CD4 counts <200 after November 2003 [19] and to all infants from 6 weeks to 12 months of age. At the time the study was started, PMTCT programs using single-dose nevirapine prophylaxis were only beginning to be established [17] and infant formula was not provided as part of these programs. Given the disadvantaged economic circumstances of the communities surrounding the two clinics and the high rates of infectious disease morbidity and mortality among children in these communities, replacement feeding was not deemed a safe alternative. All women signed informed consent. The study was approved by the Human Subjects Committees at all the investigators' institutions (Columbia University, University of Zambia, Boston University, Children's Hospital of Los Angeles).

### Study Intervention

All women, regardless of their random assignment, were encouraged to breastfeed exclusively to 4 months. Counseling to support EBF began at antenatal visits and included education about HIV transmission reduction. Study counselors were trained using a modification of the WHO breastfeeding training program [20]. Orientation sessions were provided to non-study clinical staff about benefits of EBF. Both clinics had “baby-friendly” policies [21] in place in their maternity units prior to the study. Educational messages about EBF were included in community education and outreach activities, including community drama presentations and talks. Nurse midwives attending deliveries, even if not study staff, were trained to support initiation of breastfeeding as soon as possible after delivery. A home visit scheduled 4 days after delivery focused on preventing early problems with breastfeeding initiation. Clinic-based counseling sessions every month post-partum were conducted by study counselors. Clinic visits were interspersed between home visits so that contact with the participant occurred every 2 weeks, up to 6 months of age. In addition, study participants organized themselves into “mothers' support groups” who engaged in various income-generating and self-help activities. The benefits of EBF was a unifying theme that served to motivate these groups. Infant formula was not provided before 4 months barring exceptional circumstances e.g. maternal death, desertion or severe illness.

Participants randomized to the intervention group were encouraged to breastfeed exclusively to 4 months and then to stop breastfeeding abruptly, or as rapidly as possible. Infant formula and weaning cereal were provided to women in this group at 4 months if they elected to adhere with their random assignment. Participants randomized to the control group were encouraged to exclusively breastfeed to 6 months and then to gradually introduce complementary foods (not provided by the study) while continuing to breastfeed. The duration of breastfeeding in this group was based on the women's informed choice. Women were told their random assignment usually around 2 months post-partum.

### Study procedures

Blood samples were drawn from women at enrollment during pregnancy. The results of the routine syphilis screening test (RPR) were recorded and all women with positive results were treated with penicillin. Questionnaires collected socio-economic and clinical history data at enrollment. Obstetric and neonatal data were collected at delivery. Infant heelstick blood samples were collected onto filter paper on the day of birth, at 1 week, and at 1, 2, 3, 4, 4 ½, 5, 6, 9, 12, 15, 18, 21, and 24 months of age. Clinic visits scheduled at these same time-points included a structured questionnaire about infant feeding practices. Questions included 24 hour, one week and since the last visit recall about infant consumption of any non-breast milk substances (classified as non-human milks, non-milk liquids, solids and semi-solids, fermented products, medicines, and other). Those administering the questionnaire were separate individuals to those performing the counseling and were trained to ask each question, not conditional on global screening questions. Questions were asked about the frequency of feeding all non-breast milk items and reasons for any non-EBF. The questionnaire modified an earlier version developed by WHO [22]. As a separate assessment, counselors recorded their opinion following the counseling session as to whether breastfeeding was exclusive or not.

### Laboratory methods

Maternal blood collected at enrollment was tested for CD4 and CD8 counts (FACSCount system, BD Immunocytometry Systems, San Jose, CA), hemoglobin (Hemocue® system, Lake Forest, CA) and plasma viral load (Roche Amplicor® 1.5, Branchburg, NJ). Infant heelstick samples were tested in batches for HIV-1 DNA by realtime PCR. All positives were confirmed on at least 2 samples if available. If the child died or no further samples were available, then the same sample was re-tested to confirm. All samples were confirmed to have adequate material by amplification of the beta-globin gene to minimize false negative results due to inadequate samples.

### Statistical methods

For the primary analysis, all randomized mother-infant pairs in either the intervention and control group were combined. In the case of multiple surviving infants per woman, the first-born twin was selected. HIV transmission was treated as a time-to-event variable using Kaplan-Meier methods to estimate the cumulative probability of testing HIV positive by 4 months of age (133 days). Non-EBF was treated as a time-dependent covariate which changed from EBF to non-EBF irreversibly the first time anything other than breast milk was reported on the structured questionnaire. Prescribed medicines were allowed but all other non-breast milk substances (including water) given within the past 24 hours or at least once per week in the past week or since the last visit prompted a transition into the non-EBF category. The age non-EBF started was imputed as 3 days, 10 days and 14 days prior to the actual dates of the 1 week, 1 month and 2, 3, 4 month visits respectively. Cox Proportional Hazards models were used to investigate confounding as follows: characteristics were screened as potentially associated with postnatal transmission to 4 months, those associated with transmission (p<0.10) were included one-by-one with non-EBF and if either significantly associated with transmission (p<0.05) or if they changed the estimate of the relative hazard (RH) associated with non-EBF by >10% were retained. The final Cox model included all variables that met these criteria when simultaneously entered. Differences in other characteristics were tested using chi-squared tests if these characteristics were categorical, t-tests if normally-distributed continuous and Wilcoxon rank sum tests if non-normal continuous variables. To investigate age-specific hazard rates of postnatal transmission, actuarial life-table methods were used. Event time was censored at the age (in days) when all breastfeeding ended plus 42 days or at the time of first positive test. Event time was divided by 30.417 to make months the unit of analysis. The failure probability and the hazard rate were estimated for intervals of 4 months through 24 months. Analysis used SAS software version 9.3.1 (Cary, NC).

## Results

### Study population

The study randomized 958 live-born infants of HIV-infected mothers. For this analysis, 56 (5.8%) were excluded because they had a positive result ≤3 days (presumed intrauterine infections); 61 (6.4%) were excluded because they had a positive result by 6 weeks (presumed intrapartum infections); 11 (1.1%) died by 6 weeks of age without a positive HIV result (6 died with positive results); and 55 (5.7%) were lost to follow-up or had withdrawn by 6 weeks. We also excluded 41 (5.3%) children known to have stopped breastfeeding before 4 months. The analysis includes 734 infants surviving HIV-free to 6 weeks still breastfeeding at 4 months.

### Uptake of EBF

Uptake of EBF was excellent. Viewed cross-sectionally, >94% of women reported EBF at each clinic visit. Viewed longitudinally, 613 (83.5%) of women still breastfeeding at 4 months reported giving the infant only breast milk up to that point. Non-human milk was the most commonly given item, followed by other non-milk liquids. Semi-solids were rarely given. Among those who reported non-EBF, just under half reported non-EBF in the prior 24 hours. The counselors' opinion (following the counseling session) was a poor predictor of whether non-EBF would be reported in the structured questionnaire (Table 1).

**Table 1:** Infant feeding practices reported at each clinic visit through 4 months among 734 HIV-infected mothers and their infants surviving HIV-free to 6 weeks of age and still breastfeeding to 4 months in Lusaka, Zambia.

	1 week	1 month	2 months	3 months	4 months
	N = 693	N = 685	N = 678	N = 672	N = 646
N (%) Any report of non-exclusive breastfeeding*	43 (6.2)	29 (4.2)	14 (2.1)	22 (3.3)	32 (5.0)
N (%) given:†					
Non-human milk	10 (1.4)	4 (0.6)	3 (0.4)	7 (1.0)	6 (0.9)
Semi-solids	1 (0.1)	1 (0.2)	3 (0.4)	6 (0.9)	11 (1.7)
Non-milk liquids	6 (0.9)	4 (0.6)	3 (0.4)	5 (0.7)	4 (0.6)
Water	9 (1.3)	5 (0.7)	4 (0.6)	5 (0.7)	10 (1.6)
Other‡	20 (2.9)	16 (2.3)	5 (0.7)	6 (0.9)	10 (1.6)
N (%) frequency:					
In past 24 hours	18 (41.9)	10 (34.5)	7 (50.0)	9 (42.9)	16 (50.0)
5–7 times per week	2 (4.7)	5 (17.2)	2 (14.3)	2 (9.5)	4 (12.5)
2–4 times per week	6 (13.9)	9 (31.0)	0	2 (9.5)	6 (18.8)
Once per week	17 (39.5)	5 (17.2)	5 (35.7)	8 (38.1)	6 (18.8)
Sensitivity/Specificity of counselors' opinion§	0.73/0.98	0.50/0.98	0.53/0.98	0.67/0.98	0.44/0.98

* Defined as any non-breast milk liquid or solid (including water) except prescribed medicines reported as having been given in the previous 24 hours or at least once per week in the past week or since the last clinic visit.

† Given in the past 24 hours or at least once per week. More than one item could be given.

‡ Other included cooking oil, castor oil, and herbal medicines

§ Sensitivity and specificity of the counselors' opinion was calculated assuming that the self-reports of non-exclusive breastfeeding on the standardized questionnaire were the “gold standard.”

Women who reported non-EBF before 4 months were significantly more likely to be primiparous, report going out of the home without the child, be single, have a full-time job, have a water source inside their home or on their property, have a fridge, and to have delivered at the tertiary hospital. There were no significant differences in markers of HIV disease severity between EBF and non-EBF women (Table 2).

**Table 2:** Characteristics of the 734 HIV-infected women and their infants by whether or not they practiced exclusive breastfeeding (EBF) to 4 months

Characteristic*	EBF n = 631	Non-EBF n = 121	p-value
	N (%)	N (%)	
Mean age (years)	26.5	25.8	0.15
Parity:			
First child	68 (11.1)	25 (20.7)	
2^nd^–3^rd^	417 (68.0)	72 (59.5)	
≥Fourth	128 (20.9)	24 (19.8)	0.02
Median duration (months) of breastfeeding for last child‡	18	19	0.17
EBF ≥4 months for last child‡	393 (72.2)	61 (63.5)	0.09
One or more children have died‡	216 (40.4)	47 (47.0)	0.12
Disclosed HIV status to partner	351 (57.3)	68 (56.2)	0.83
Reports 0–4 months never gone out of home without child	341 (55.6)	49 (40.5)	0.002
Married	529 (86.3)	97 (80.2)	
Single	50 (8.2)	19 (15.7)	
Widowed/divorced/separated	34 (5.5)	5 (4.1)	0.03
No school	33 (5.4)	4 (3.3)	
Primary school (<8 years)	309 (50.4)	63 (52.1)	
Some high school (≥8 years)	222 (36.2)	42 (34.7)	
High school completed or more	49 (8.0)	12 (9.9)	0.70
Tap in dwelling or on own property	97 (15.8)	30 (24.8)	
Community tap or other	516 (84.2)	91 (75.2)	0.02
Electricity in the home	238 (38.8)	54 (44.6)	0.23
Cooking:			
Stove/hotplate	207 (33.8)	50 (41.3)	
Charcoal/wood	405 (66.2)	71 (58.7)	0.11
Fridge in the home	71 (11.6)	23 (19.2)	0.02
Report ≥1 day in past month no food at home	143 (23.3)	27 (22.3)	0.81
Full-time paid job	36 (5.9)	14 (11.6)	0.02
Place of birth:			
Clinic	473 (77.2)	86 (71.1)	
Home	64 (10.4)	7 (5.8)	
Hospital	72 (11.8)	25 (20.7)	
Other	4 (0.6)	3 (2.5)	0.006
Infant sex:			
Male	330 (53.9)	57 (47.1)	
Female	282 (46.1)	64 (52.9)	0.17
Breastfeeding initiated within:			
≤30 min	391 (64.4)	76 (64.4)	
30–60 min	166 (27.4)	28 (23.7)	
>60 min of delivery	50 (8.2)	14 (11.9)	0.38
Mode of delivery:			
Cesarean	10 (1.6)	6 (5.0)	0.02
Mean birth weight (grams)	3010	3068	0.29
Infant Birth weight			
<2,500 grams	58 (9.5)	12 (9.9)	0.88
Median maternal CD4 count	354	327	0.57
<200	144 (23.5)	30 (24.8)	
200–349	164 (26.7)	26 (21.5)	
350–499	185 (30.2)	36 (29.7)	
≥500 cells/mm^3^	120 (19.6)	29 (24.0)	0.55
Median maternal plasma viral load	29,482	43,989	0.08
<1000	49 (8.0)	9 (7.4)	
1,000–9,999	132 (21.6)	20 (16.5)	
10,000–99,999	283 (46.3)	55 (45.5)	
≥100,000 copies/ml	147 (24.1)	37 (30.6)	0.39
Hemoglobin			
<10 g/dL	151 (25.0)	36 (30.3)	0.23
RPR positive	108 (18.6)	21 (19.6)	0.81
Body mass index 1 month post-partum			
<18.5	86 (14.1)	17 (14.1)	0.99
Experimental group	300 (48.9)	69 (57.0)	
Control group	313 (51.1)	52 (43.0)	0.10

* Data shown are the number (percentage) with each characteristic within the assigned group (unless otherwise indicated e.g. mean/median). Maternal CD4 counts, viral load, hemoglobin, RPR status and eligibility for antiretroviral therapy were determined based on blood samples and clinical history collected at enrollment during pregnancy; social and economic characteristics were also measured at this time.

‡ Only among those who reported one or more previous liveborn children

### Reduced HIV transmission with EBF

**Figure 1:**
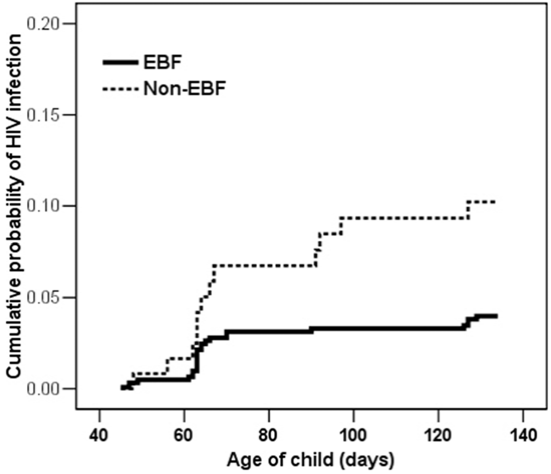
Cumulative probability of testing HIV positive by PCR through 4 months among 613 exclusively breastfed (EBF) infants and 121 non-exclusively breastfed (Non-EBF) infants born to HIV-infected women in Lusaka, Zambia. Non-EBF was defined as giving the child any non-breast milk items (except prescribed medicines) in the past 24 hours or at least once per week at any time before 4 months.

The risk of post-natal HIV transmission before 4 months was significantly lower among 613 women who reported EBF (cumulative probability of a positive PCR by 4 months 0.040 [n = 24] 95% CI: 0.024–0.055) compared to among 121 who reported non-EBF (0.102 [n = 12] 95% CI: 0.047–0.157) (p = 0.004) (Figure 1). In unadjusted analysis, non-EBF (defined as time-dependent covariate) was associated with >3-fold increased risk of early postnatal HIV transmission (RH = 3.48 95% CI: 1.71–7.08) (Table 3). The risk was slightly increased (RH = 4.04 95% CI: 1.87–8.71) if the analysis was restricted to those 727 pairs with negative PCR results >28 days (a more stringent analysis ensuring that infections detected >6 weeks were not due to intrapartum infections detected late).

**Table 3:** Risk factors for early postnatal HIV transmission ≤4 months of age among 734 infants born to HIV-infected women in Lusaka, Zambia

	N total	N infected	Probability of infection	Unadjusted relative hazard (95% CI)	Adjusted relative hazard (95% CI)¶
Exclusive breastfeeding	613	24	0.0398		
Non-exclusive breastfeeding	121	12	0.1022	3.48 (1.71–7.08)*	2.68 (1.27–5.62)
Maternal CD4 counts					
>350	364	5	0.0141		
<350	370	31	0.0855	6.33 (2.46–16.26)	3.19 (1.19–8.55)
Maternal plasma viral load					
<10,000	210	2	0.0096		
10,000–100,000	338	11	0.0332		
>100,000 copies/ml	184	23	0.1284	4.02 (2.28–7.10)†	3.24 (1.71–6.11)
Maternal hemoglobin					
>10 g/dL	536	20	0.0380		
<10 g/dL	187	16	0.0872	2.32 (1.20–4.48)	
RPR status during pregnancy					
Negative	558	23	0.0422		
Positive	129	11	0.0870	2.14 (1.04–4.39)	2.17 (1.05–4.51)‡
Infant sex					
Male	387	19	0.0498		
Female	346	17	0.0505	1.00 (0.52–1.93)	
Birthweight					
>2500 g	649	29	0.0456		
<2500 g	70	6	0.0872	1.98 (0.82–4.77)	1.14 (0.46–2.81)§
Experimental group	369	18	0.0499		
Control group	365	18	0.0502	1.01 (0.52–1.93)	

* Non-exclusive breastfeeding coded as a time-dependent covariate

† Maternal plasma viral load coded as a continuous variable indicating transmission risk per log10 increase

‡ A dummy variable for missing RPR status was included in the adjusted model to avoid excluding those missing their RPR status data (n = 47)

§ Low birthweight was included in the adjusted model because its inclusion reduced the magnitude of the association between non-exclusive breastfeeding and transmission

¶ The adjusted model included simultaneously the five covariates displayed in the final column.

The questionnaire asked about why breastfeeding was not exclusive. The most common reason was that the infant was either crying or sick (n = 41). If these pairs were excluded, there continued to be a significant association between non-EBF and transmission (RH = 2.92 95% CI: 1.19–7.14). The other reasons given for non-EBF were that the child was cared for by others (n = 24), the mother was advised to do so (n = 14), insufficient milk (n = 6), to relieve child constipation (n = 3), other (n = 5) and no reason given or denies non-EBF (n = 28).

We examined the consequences of defining non-EBF in different ways. If non-EBF was defined as giving the child non-human milk, semi-solids or non-milk liquids only (i.e. allowing water or “other” items to be given) in the last 24 hours or at least once per week, then the association with transmission was slightly stronger (RH = 5.56 95% CI: 2.43–12.74). Giving water or “other” substances was not associated with a significant increase in transmission (RH = 1.87 95% CI: 0.73–4.81).

We also examined associations by the regularity of non-EBF. Surprisingly, we found stronger associations with increased transmission with less regular relative to more regular non-EBF. If non-EBF was defined as giving the child any non-breast milk items 5 or more times per week or within the past 24 hours, the association with transmission was weaker (RH = 2.04 95% CI: 0.72–5.78) than if non-EBF was defined as giving any non-breast milk items less regularly i.e. 1–4 times per week and not in the last 24 hours (RH = 4.12 95% CI: 1.93–8.79).

To investigate if differences between the women who practiced non-EBF explained the association, we conducted multivariate analyses adjusting for other risk factors for early postnatal transmission. The association between non-EBF and higher risk of post-natal HIV transmission was slightly attenuated but remained significant (RH = 2.68 95% CI: 1.28–5.62) after adjusting for maternal CD4 count, plasma viral load, syphilis screening results and low birthweight (Table 3). None of the socioeconomic or other characteristics that differed between women reporting EBF or non-EBF were associated with postnatal transmission, nor did their inclusion influence the EBF-transmission association.

Maternal CD4 count was a strong predictor of early post-natal transmission with 86.1% of early postnatal infections occurring among women with CD4 counts <350. Among women with CD4 counts <350, the cumulative probability of postnatal HIV transmission by 4 months was 0.191 (95% CI: 0.095–0.288) for non-EBF (n = 65) and 0.063 (95% CI: 0.036–0.091) for EBF (n = 306); RH = 3.40 (95% CI: 1.58–7.33) after adjusting for viral load, syphilis status and low birthweight. Among women with CD4 counts >350, there were no infections by 4 months for non-EBF (n = 56) and the cumulative probability of postnatal transmission was 0.017 (95% CI: 0.002–0.031) for EBF (n = 307).

Six women started on antiretroviral therapy during pregnancy. If these women are excluded and person-time censored when an additional 3 women started on therapy after delivery, the association between non-EBF and transmission remained unchanged (RH = 3.09 95% CI 1.25–7.64 adjusted for viral load, CD4 count, syphilis status and birthweight).

### Reverse causality

To investigate reverse causality i.e. child HIV infection increasing the likelihood of non-EBF, rather than non-EBF increasing the likelihood of HIV infection, we examined the association between EBF and intrauterine and intrapartum-acquired infections. These two routes of infection cannot be influenced by postnatal circumstances. Defining non-EBF as giving any non-breast milk item to the infant before 4 months, there was no association with intrauterine infection (RH = 0.83 95% CI 0.42–1.65) or intrapartum infection (RH = 1.18 95% CI: 0.65–2.14). If the definition of non-EBF was revised to be non-EBF before 1 month of age, there remained no association with intrauterine transmission (RH = 1.09 95% CI: 0.50–2.42), but there was a slight, non-significant, increase in intrapartum transmission (RH = 1.75 95% CI: 0.89–3.44) suggesting a small proportion of postnatal transmission may have been misclassified as intrapartum.

### Postnatal transmission older than 4 months

As an alternative approach to considering the role of non-EBF in HIV transmission, we investigated age-specific hazard rates of postnatal transmission over 4 month intervals using actuarial life-table methods. Since the design of the study specifically attempted to stop all breastfeeding at 4 months in the intervention group, we only included post-4 month data from the control group. The median duration of breastfeeding in the control group was 16 months (interquartile range 11–19 months). All women in the control group were encouraged to introduce complementary foods by 6 months. Therefore early transmission is predominantly EBF (>80% reported only EBF) whereas the later transmission is all non-EBF (all had introduced complementary foods by 6 months). Although the proportion exclusively breastfeeding declined to zero at older child ages, the hazard of postnatal transmission (instantaneous probability of HIV infection per month of breastfeeding) did not increase with child age. Rather, in the first 4 months, the overall hazard rate was 0.0107 (∼1.1% per month) (95% CI: 0.0052–0.0161). In the intervals 5–8 months and 9–12 months, the hazard rate remained in the 1% per month range. After 12 months, it declined to ∼0.5% per month over the 12–24 month period (hazard = 0.0052 95% CI: 0.0014–0.0091).

**Figure 2:**
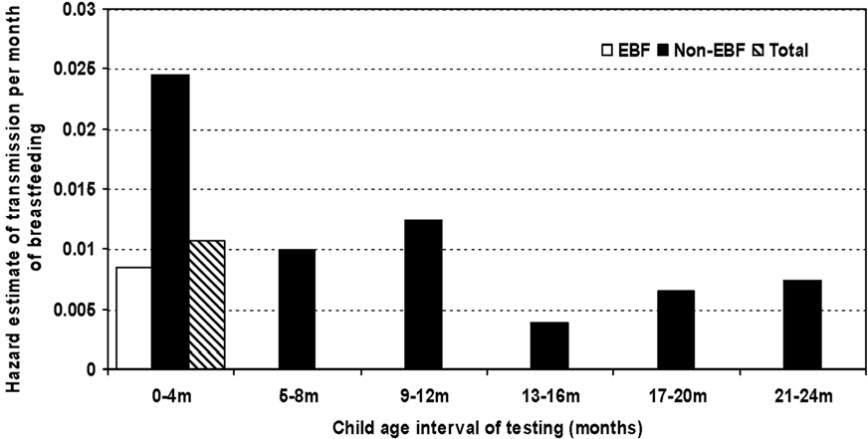
Hazard rates of breastfeeding HIV transmission per month averaged over 4 month intervals through 24 months

For comparison using this method, the monthly hazard in the first 4 months was <1% among the EBF group (0.0085 95% CI: 0.0048–0.0122) compared to 2.4% per month among the non-EBF over this period (0.0244 95% CI: 0.0100–0.0389). Thus, there was a significant decline in the hazard of postnatal *non-EBF* HIV transmission from ∼2.4% per month during the first 4 months, declining to ∼1% per month over the interval 5–12 months, and ∼0.5% per month after 12 months (Figure 2, Table 4).

**Table 4:** Actuarial life-table estimates of HIV transmission hazard rates per breast feeding month in the control group (n = 365)

	Age interval in months					
	0–4	5–8	9–12	13–16	17–20	21–24
No. infections in age interval	15	12	12	3	3	1
Hazard rate per month in interval	0.0107	0.00995	0.01237	0.0039	0.0066	0.0074
95% CI	0.0052–0.0161	0.0043–0.0155	0.0053–0.0194	0–0.0083	0–0.0140	0–0.0218

## Discussion

Our data demonstrate that infants of HIV-infected mothers who are breastfed exclusively to 4 months are at least 50% less likely to acquire HIV infection through breastfeeding. The reduction in HIV transmission associated with EBF remained significant after adjustment for both maternal CD4 count and viral load. This enabled us to largely rule out the objection that the association is due to sicker women self-selecting non-EBF. Our findings also strongly argue that reverse causality (i.e. illness among infected children prompting non-EBF behaviors, rather than non-EBF being a cause of infection) does not explain the association since we observed no association between non-EBF and intrauterine/intrapartum transmission.

Discussions of possible mechanisms underlying the benefits of EBF for HIV transmission have focused on inflammation or activation within the infant gut as a result of the introduction of foreign antigens, contaminants and pathogens [9], [10], [14], [15], [23]. We speculate that there may be additional processes including elevations in breast milk vial load as a result of decreased frequency of infant suckling. Since the frequency of milk removal dictates the rate of milk secretion [24], small changes, such as a missed feed or an irritable infant not suckling as much as usual, may result in some milk stasis (breast engorgement). If not reversed within a short period of time, epithelial permeability may increase (leaky tight junctions) [25], [26], allowing more efficient paracellular transfer of both cell-free and cell-associated HIV [25]. In a prior report, we demonstrated that breast milk HIV RNA concentrations increased dramatically following abrupt weaning [27]. We also reported here, intriguingly, that irregular, rather than regular, use of non-breast milk items was associated with stronger associations with transmission. We speculate that the shifts in breast feeding frequency that occur with non-EBF, particularly if it is irregular, may disrupt the efficiency of milk removal and may increase the infectivity of breast milk.

Our counseling program encouraged EBF in conjunction with other standard messages related to lactation support, including education about initiation of breastfeeding immediately after delivery, correct breastfeeding techniques, frequent on-demand feeding, and prompt management of breast problems. Counseling to support lactation reduces the risk of mastitis [28], which is a strong risk factor for HIV transmission [29], [30], and it is likely that the benefits of EBF for HIV prevention may depend on these other dimensions of healthy breastfeeding.

Our findings of reduced HIV transmission with EBF are consistent with at least three other large African studies [9], [10], [14], [15]. Our estimate of the magnitude of the reduction is slightly less than reported in these prior studies. This may be because we used a stringent EBF definition that allowed no occurrence of any non-breast milk item; the reduction was stronger if water and “other” items were allowed. The smaller magnitude may also be because few women gave their infants semi-solids which has been found to convey the highest risks of postnatal HIV transmission [15]. Thus the avoidance of infant formula and semi-solids may be most crucial in the early months of a breast-fed infant's life to reduce risks of HIV transmission.

Our study of EBF reported here was an observational sub-study nested within a clinical trial designed to evaluate whether stopping all breastfeeding at 4 months improves HIV-free survival of infants born to HIV-infected mothers. The clinical trial demonstrated no benefit for HIV-free survival of stopping breastfeeding at 4 months compared to continuation to a median of 16 months [31]. The rationale for stopping breastfeeding at 4 months was to avoid any period of non-EBF since introduction of complementary foods was recommended at 4–6 months at the time the trial was designed [32]. Our final results demonstrated that the supposition that all non-EBF should be avoided was incorrect [31]. Rather, as we demonstrate here, the risk of post-natal HIV transmission occurring during non-EBF is greater in the first 4 months of life and declines as the child becomes older. Non-EBF i.e. continuing breastfeeding with complementary foods, is developmentally-appropriate at older ages and conveys less of a risk of HIV transmission than non-EBF in the first few months of life. The biological basis for this distinction is not clear but our results are broadly consistent with several previous studies that have reported either constant or declining transmission hazards with age [33]–[38]. The prior studies are difficult to compare directly since EBF and non-EBF are not distinguished and feeding practices are likely to differ substantially across sites as well as over time as different recommendations about infant feeding by HIV-infected women have been implemented. The net result is that benefits of reducing postnatal HIV transmission through early cessation of breastfeeding are too small to offset the high competing risks of mortality attributable to the absence of breast milk in the diet of an infant between 4 and 24 months.

Uptake of EBF was excellent in our study with more than 80% of women reporting only EBF to 4 months. We attribute this high uptake to the multidimensional intervention which included intensive and skilled counseling, consistent messages across the health service, establishment of a mothers' lactation support network and active community outreach promoting health benefits of EBF for all women regardless of their HIV status. EBF concepts were not new at the study sites since “baby friendly” policies had been part of Zambian guidelines before the study began. Despite these policies, the demographic health survey in 2001–2002 estimated that 45% of 2–3 month old infants in Zambia were exclusively breastfed, declining to 15% by 4–5 months [39]. More research is needed regarding how to achieve such high uptake of EBF among HIV-infected women in programmatic settings but our data are encouraging that EBF can be achieved among a high proportion of HIV-infected women with a modest intervention in the context of clinical research.

There are several prior studies that have investigated health services and community-based interventions to improve in EBF uptake among uninfected women [40]–[42]. These studies should be extended to populations with substantial burdens of HIV infection. Knowledge of the capacity of EBF to reduce HIV transmission may in fact increase motivation for this practice. Special attention may be needed for young primiparous mothers and for women working in the formal sector since these were barriers to EBF in our study, as is often observed [43]. Further qualitative and social science research may be helpful to identifying how to implement effective programs.

In conclusion, we have demonstrated that non-EBF more than doubles the risk of postnatal HIV transmission in the first 4 months of life. A 50% reduction in postnatal HIV transmission with a low cost and sustainable intervention has major public health implications and a reduction of this magnitude is within the range of many antiretroviral interventions. Given the important health benefits of EBF, it is critical that further research be undertaken to investigate how to achieve good uptake of EBF in the populations most affected by the HIV pandemic. This is essential so that effective programs can be implemented as integral components of PMTCT and antiretroviral treatment programs in low resource settings. EBF is a safe and effective practice that substantially helps HIV-infected women protect their children's lives. We also encourage strengthening lactation support programs in antenatal, maternity and well-baby services in the general population to benefit uninfected mothers and their children as well.

## Supporting Information

Protocol S1 Trial protocol
(0.12 MB DOC)
